# Importance and Antimicrobial Resistance of *Mycoplasma bovis* in Clinical Respiratory Disease in Feedlot Calves

**DOI:** 10.3390/ani11051470

**Published:** 2021-05-20

**Authors:** Ana García-Galán, Juan Seva, Ángel Gómez-Martín, Joaquín Ortega, Francisco Rodríguez, Ángel García-Muñoz, Christian De la Fe

**Affiliations:** 1 Ruminant Health Research Group, Department of Animal Health, Faculty of Veterinary Sciences, Regional Campus of International Excellence “Campus Mare Nostrum”, University of Murcia, 30100 Murcia, Spain; 2 Department of Anatomy and Comparative Pathological Anatomy, Faculty of Veterinary Sciences, Regional Campus of International Excellence “Campus Mare Nostrum”, University of Murcia, 30100 Murcia, Spain; jseva@um.es; 3 Microbiological Agents Associated with Animal Reproduction (ProVaginBIO) Research Group, Departamento Producción y Sanidad Animal, Salud Pública Veterinaria y Ciencia y Tecnología de los Alimentos, Facultad de Veterinaria, Universidad Cardenal Herrera-CEU, CEU Universities, 46113 Valencia, Spain; angel.gomezmartin@uchceu.es; 4 Pathology Group, PASAPTA, Facultad de Veterinaria, Universidad Cardenal Herrera-CEU, CEU Universities, Av. Seminario s/n, 46113 Valencia, Spain; jortega@uchceu.es; 5 Unit of Veterinary Histology and Pathology, Institute for Animal Health, Veterinary School, University of Las Palmas de Gran Canaria, 35413 Gran Canaria, Spain; francisco.guisado@ulpgc.es; 6 Departamento Producción y Sanidad Animal, Salud Pública Veterinaria y Ciencia y Tecnología de los Alimentos, Facultad de Veterinaria, Universidad Cardenal Herrera-CEU, CEU Universities, 46113 Valencia, Spain; angel@uchceu.es

**Keywords:** *Mycoplasma bovis*, pneumonia, antimicrobial resistance, feedlot calves, minimum inhibitory concentration

## Abstract

**Simple Summary:**

Bovine respiratory disease is a common health and economic problem that mainly affects calves raised in feedlots. Several viruses and bacteria may be involved, but *Mycoplasma bovis* can cause disease chronification and poor response to antimicrobial treatment. This study investigated the role of *Mycoplasma* *bovis* in cases of clinical respiratory disease unresponsive to treatment that affected feedlot calves in southeast Spain, and tested the in vitro susceptibility of a selection of isolates to the specific set of antimicrobials used for therapy in vivo. *Mycoplasma bovis* was found in 86.9% (20/23) of the calves, predominantly in the lungs (78.26%; 18/23) where it was involved in pulmonary lesions. Furthermore, the selected isolates were found to be resistant in vitro to most of the antimicrobials specifically used for treating the animals in vivo. These results highlight the implication of *Mycoplasma bovis* in the bovine respiratory disease affecting feedlot calves in Spain.

**Abstract:**

Bovine respiratory disease (BRD) is an important viral and/or bacterial disease that mainly affects feedlot calves. The involvement of *Mycoplasma bovis* in BRD can lead to chronic pneumonia poorly responsive to antimicrobial treatment. Caseonecrotic bronchopneumonia is a pulmonary lesion typically associated with *M. bovis*. In Spain, *M. bovis* is widely distributed in the feedlots and circulating isolates are resistant to most antimicrobials in vitro. However, the role of this species in clinical respiratory disease of feedlot calves remains unknown. Furthermore, available data are relative to a fixed panel of antimicrobials commonly used to treat BRD, but not to the specific set of antimicrobials that have been used for treating each animal. This study examined 23 feedlot calves raised in southeast Spain (2016–2019) with clinical signs of respiratory disease unresponsive to treatment. The presence of *M. bovis* was investigated through bacteriology (culture and subsequent PCR), histopathology and immunohistochemistry. The pathogen was found in 86.9% (20/23) of the calves, mainly in the lungs (78.26%; 18/23). Immunohistochemistry revealed *M. bovis* antigens in 73.9% (17/23) of the calves in which caseonecrotic bronchopneumonia was the most frequent lesion (16/17). Minimum inhibitory concentration assays confirmed the resistance of a selection of 12 isolates to most of the antimicrobials specifically used for treating the animals in vivo. These results stress the importance of *M. bovis* in the BRD affecting feedlot calves in Spain.

## 1. Introduction

Bovine respiratory disease (BRD) is a major disease of feedlot cattle that affects the upper or lower respiratory tract, causing high mortality and carcasses of lower quality [1]. Typical clinical symptoms include fever, dyspnea, coughing, nasal or eye discharge, depression and decreased or no appetite [2]. BRD has a multifactorial etiology, including infectious agents, host predisposing factors and environmental stressors. The disease usually appears in calves after a stressful environmental event such as weaning, transportation, co-mingling or drastic diet or weather changes [3]. Infectious agents commonly associated with BRD are the viruses bovine viral diarrhea virus (BVDV), bovine herpesvirus type 1 (BHV-1), parainfluenza-3 (PI-3) virus, bovine coronavirus (BCOV) and bovine respiratory syncytial virus (BRSV), and the bacteria *Pastereulla multocida, Mannheimia haemolytica*, *Histophilus somni*, *Trueperella pyogenes* and *Mycoplasma bovis* [4,5,6]. Generally, viruses are considered to be primarily BRD initiators that then promote colonization by bacterial pathogens [7]. Among bacteria, *M. bovis* can also act as a primary pathogen and is recognized as an important cause of chronic pneumonia that seems poorly responsive to antimicrobial treatment [7,8].

Often, calves become infected with *M. bovis* by close contact with asymptomatic carriers, which are occasionally shedding the pathogen in colostrum, milk, nasal or genital secretions [9,10,11]. Some animals acquire the infection at the farm of origin and others become infected once arrived at the feedlot [12,13,14]. In northern Italy and northwestern Spain, the analyses of pneumonic lungs recovered from beef cattle with subclinical pneumonia revealed the presence of the pathogen in 25 (16/64) and 66% (33/50) of the animals [5,15]. In eastern and western France, *M. bovis* was isolated in 78.5 (106/135) and 52.1% (60/115) of the feedlot calves at the onset of BRD outbreaks, based on the analyses of broncho-alveolar lavage (BAL) and nasal swab samples, respectively [4,16].

Caseonecrotic bronchopneumonia with multiple foci of caseous necrosis is a pulmonary finding typically associated with *M. bovis* in naturally or experimentally infected calves [8,17,18]. Other authors consider the bronchiolitis as another distinctive lesion [19,20,21]. Furthermore, the pathogen may be involved in other lesions such as necrosis of the bronchiolar epithelium, bronchus-associated lymphoid tissue (BALT) hyperplasia, and bronchiolar fibrosis [19,20,22]. Additionally, *M. bovis* may be involved in lesions such as bronchopneumonia with foci of coagulative necrosis or abscesses, but usually in co-infection with other bacteria [12]. In such cases, the necrosis lesions originated from bronchioles or small bronchi are distinctive of the mycoplasma presence [22]. In addition to the histopathological analyses of pneumonic lesions, the presence of *M. bovis* has to be confirmed by bacteriological (culture and subsequent PCR) and/or immunohistochemical analyses [18,22].

Prevention and control of *M. bovis* pneumonia mainly rely on antimicrobial treatment as there are no efficient vaccines available [23]. However, in vitro antimicrobial resistance has been reported by many countries worldwide [24,25]. In Spain, recent studies have demonstrated the extended circulation of *M. bovis* in beef cattle herds and the circulation of isolates resistant to most antimicrobials in vitro [26,27,28]. For minimum inhibitory concentration (MIC) assays, those studies included some isolates from the lungs of young animals with clinical respiratory disease, but pulmonary lesions compatible with *M. bovis* were not evaluated. Hence, the role of *M. bovis* in the clinical respiratory disease of feedlots calves in Spain remains to be addressed. On the other hand, these studies tested the in vitro susceptibility of *M. bovis* isolates against a set of antimicrobials commonly used in the field, but did not consider the specific antimicrobials used for the treatment of each animal in vivo.

This study was conducted (i) to address the role of *M. bovis* in clinical respiratory disease unresponsive to antimicrobials in feedlot calves in Spain through bacteriological, histopathological and immunohistochemical techniques; and (ii) to determine the MIC values of the isolates recovered against the specific set of antimicrobials used for therapy in vivo.

## 2. Materials and Methods

### 2.1. Animal Sampling

All animal procedures met the conditions set out in the EU Directive 2010/63/EU for animal experimentation and had the authorization of the Ethics Committee on Animal Testing of the University of Murcia (Number: 307/2017).

In this study, 23 calves (50–350 kg), raised in 12 feedlots placed in the southeast of Spain, were sampled over a four-year period (2016–2019). The epidemiological background of the animals is summarized in Table 1. The animals showed clinical signs of respiratory disease and did not respond to antimicrobial treatment. Most of them (18/23) were euthanized with T-61 (MSD, Kenilworth, NJ, USA) by the feedlots’ trained veterinary staff and the carcasses were submitted for necropsy. The necropsies and sampling procedures were conducted following the standard necropsy procedures for ruminants. In these animals, one nasal, auricular and conjunctival swab from the left and right side, one lung swab and one lung tissue specimen were collected (*n* = 5 per animal). The remaining five animals (5/23) were sacrificed at the slaughterhouse. In these calves, one lung swab and one lung tissue specimen were obtained (*n* = 2 per animal). In all cases, lung swabs and lung tissues were obtained from areas of cranioventral consolidation. In total, 100 samples were obtained. The sample collection was composed of auricular (*n* = 18), conjunctival (*n* = 18), nasal (*n* = 18) and lung (*n* = 23) swabs, as well as lung tissues (*n* = 23).

### 2.2. Mycoplasma Cultures, DNA Extraction and PCR

Swabs were put into a sterile tube with Aimes agar transport medium (Deltalab^®^, Barcelona, Spain) and preserved at 4 °C for culture and molecular analysis. Lung tissues were put into a sterile container and fixed in 10% neutral-buffered formalin for histopathology and immunohistochemistry (IHC).

For mycoplasma isolation, swabs were incubated at 37 °C with 5% CO2 for 24 h in 2 mL of SP4 medium [29], modified as previously described [28,30]. Cultures were purified through a membrane filter of 0.45 µm (LLG-Labware, UK) and incubated for 48 h before plating 5 µL onto SP4 agar. The plates were incubated at 37 °C with 5% CO2 and checked daily under a light microscope for the presence of mycoplasma colonies.

DNA was extracted from 200 µL of broth culture [31] and the presence of *M. bovis* was confirmed by PCR [32]. By picking single colonies, PCR-positive cultures were three times cloned and the species of the final isolate was examined again by PCR.

### 2.3. Histopathology and IHC

The formalin-fixed lung tissues were embedded in paraffin wax and cross-sectioned 4–5 μm thick with a microtome for histopathology and IHC.

For histopathology, sections were stained with hematoxylin and eosin (H-E) and examined under a light microscope. On sections with necrotic foci Gram-stain was also used.

Histological sections were analyzed to assess the presence or absence of the following changes: bronchiolar necrosis, intrabronchiolar and alveolar neutrophils, bronchiolar and alveolar fibrosis, bronchiolar and alveolar syncytial cells, foci of coagulative necrosis, abscesses, foci of caseous necrosis, alveolar and septal thrombosis, fibrinous pleuritis, pleural fibrosis, BALT hyperplasia and alveolar fibrin exudation. The presence or absence of Gram-positive and Gram-negative bacteria and areas of mineralization was analyzed in the necrotic foci.

Based on the histological findings, four pulmonary patterns were defined: (i) caseonecrotic bronchopneumonia, (ii) suppurative bronchopneumonia, (iii) fibrinous bronchopneumonia and (iv) interstitial pneumonia. Other defined lesions were bronchiolitis, necrosis of the bronchiolar epithelium, BALT hyperplasia and bronchiolar fibrosis.

The following lesions were scored by means of a semi-quantitative grading system based on the piece of sample evaluated: caseonecrotic bronchopneumonia (+ size of foci < 200 µm; ++ size of foci > 200 µm, < 2000 µm; and +++ size of foci > 2000 µm); suppurative bronchopneumonia and fibrinous bronchopneumonia (+ < 25%; ++ > 25%, < 75%; and +++ > 75%); bronchiolitis and bronchiolar fibrosis (+ mild, ++ moderate and +++ severe); interstitial pneumonia (+ presence of syncytial cells); necrosis of the bronchiolar epithelium (+ < 10%; ++ > 10%, < 50%; and +++ > 50%); BALT hyperplasia (+ 1–2 lymphoid follicles < 100 µm, ++ 1–2 lymphoid follicles > 100 and +++ > 2 lymphoid follicles).

The detection of *M. bovis* antigen was carried out by IHC on paraffin-embedded sections as previously described [17], using a rabbit polyclonal antibody (Ref. PA295) raised against whole cell antigen of *M. bovis*, diluted 1:500, and the avidin biotinylated enzyme complex (ABC) method (Vector Laboratories, Burlingame, CA, USA). Substitution of the primary antibodies with mouse non-immune serum, and lung tissue from the control calves, served as negative controls. Then, the presence or absence of *M. bovis* antigen was assessed by examining the lung sections.

### 2.4. MIC Assays

MIC values were calculated only for the antimicrobials used for the treatment in vivo and with recognized antimycoplasmic effect. MIC assays were carried out when two conditions were met: (i) the treatment received by the animal in vivo was provided by the feedlot’s veterinary staff and (ii) at least one *M. bovis* isolate was obtained from the animal. In those cases, isolates obtained from lung swabs were used for MIC assays. If no isolate was obtained from the lung swab, the assay was carried out with an isolate obtained from the nasal swab or the auricular swab.

Antimicrobials used for MIC assays included (i) the macrolides, tulathromycin (Carbosiynth, Compton, UK) and tilmicosin (Molekula, Darlington, UK), (ii) the lincosamide, lincomycin (Sigma-Aldrich, St. Louis, MO, USA), (iii) the phenicol, florfenicol (Sigma-Aldrich, St. Louis, MO, USA), (iv) the aminoglycoside, gentamicin (Sigma-Aldrich, St. Louis, MO, USA), (v) the fluoroquinolones, enrofloxacin (Fluka, Bio-Chemika, St. Louis, MO, USA) and marbofloxacin (Tokyo Chemical Industry, Chuo City, Japan) and (vi) the tetracycline, oxytetracycline (Acros Organics—Thermo Fisher Scientific, Waltham, Massachusetts, USA). Stock solutions (1 mg/mL) and two-fold dilutions were prepared in sterile distilled water. Florfenicol was dissolved in 95% ethanol dropwise before being made to the correct final volume with sterile distilled water. Fluoroquinolones were prepared as previously described [28]. A final range from 128 to 0.0625 µg/mL was studied.

Stationary-phase cultures of *M. bovis* isolates were used for MIC assays. The reference strain PG45 was used as a control. The microbroth dilution method was carried out in 96-well microtiter plates. Mycoplasma cultures and MIC assays were carried out as already described [28], and following previous recommendations [33]. All the assays were repeated twice. If the results of the repeated tests differed in only one dilution, the higher MIC value was used. If the MIC value differed in more than one dilution, a third repetition was carried out and the final MIC value was the mode of the three values. MIC values were interpreted by considering breakpoints for *M. bovis* proposed by other authors or analyzing whether mutations related to antimicrobial resistance had been described for those values [28,34]. For gentamicin and lincomycin, MIC values were interpreted according to breakpoints proposed for other mycoplasma species [33,35].

## 3. Results

### 3.1. Detection of M. bovis in Different Anatomical Sites

In this study, *M. bovis* was detected in 86.9% (20/23) of the calves and 53% (53/100) of the analyzed samples (Table 1). Among the different anatomical locations studied, the pathogen was most commonly found in the lungs (78.26%; 18/23). In these samples, IHC immunolabeled 73.9% (17/23), and culture and subsequent PCR detected 65.2% (15/23). Most animals diagnosed as *M. bovis* PCR-positive were also positive by IHC, with only one exception (animal nº 8). Other PCR-positive samples were nasal (11/18), conjunctival (6/18) and auricular swabs (4/18). Generally, animals that carried *M. bovis* in these anatomical areas also carried the pathogen in the lungs. Only two exceptions were found, the animals nº 12 and nº 14, which were identified as auricular, and conjunctival and nasal carriers, respectively (Table 1).

### 3.2. Histopathology and Detection of M. bovis Antigen by IHC

**Figure 1:**
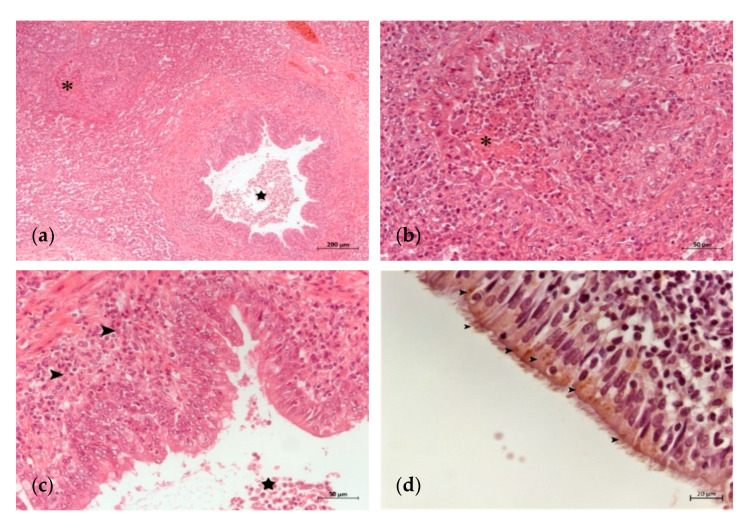
Initial histopathological lung lesions in a calf with *M. bovis* infection: (**a**) area of suppurative bronchopneumonia with inflammatory infiltrate in the wall and the lumen of a bronchiole (star) and the beginning of a caseonecrotic focus (asterisk) (H-E); (**b**) detail of the caseonecrotic focus of (**a**) associated with neutrophilic exudation (asterisk) (H-E); (**c**) bronchointerstitial lymphoplasmacytic (arrowheads) and neutrophilic (star) inflammatory infiltrate predominate in the bronchiolar wall and lumen, respectively (H-E); (**d**) detail of the bronchiolar epithelial cells showing mononuclear infiltration and *M. bovis* antigen (arrowheads) (IHC).

**Figure 2:**
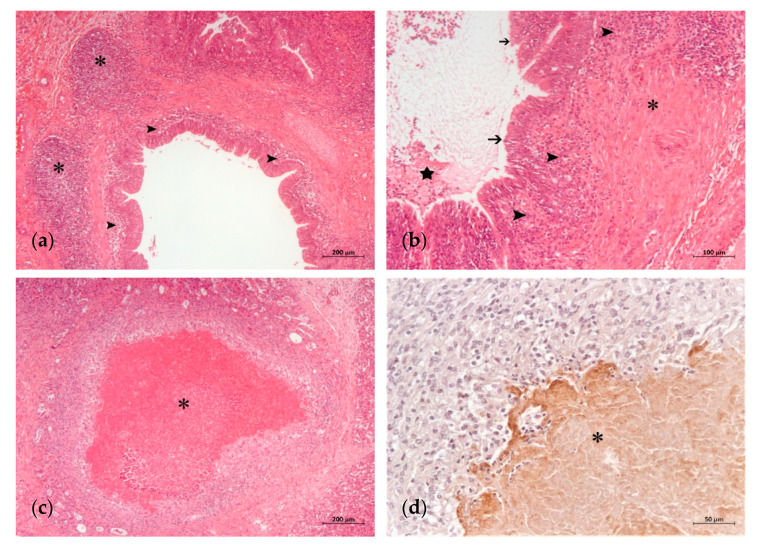
Advanced histopathological lung lesions in a calf with *M. bovis* infection: (**a**) prominent BALT hyperplasia (asterisks) and bronchitis (arrowheads) (H-E); (**b**) abundant mucopurulent exudate in the lumen of a bronchiole (star), fibrosis (asterisk) of the wall along with mononuclear inflammatory infiltrate (arrowhead) and necrosis of the epithelial surface (arrows) (H-E); (**c**) extended caseonecrotic focus (asterisk) with eosinophilic center demarcated by inflammatory cells, remnants of necrotic bronchiolar epithelium and fibrosis (H-E); (**d**) detail of caseonecrotic focus (asterisk) with granular *M. bovis* antigen predominantly at the periphery (IHC).

**Figure 3:**
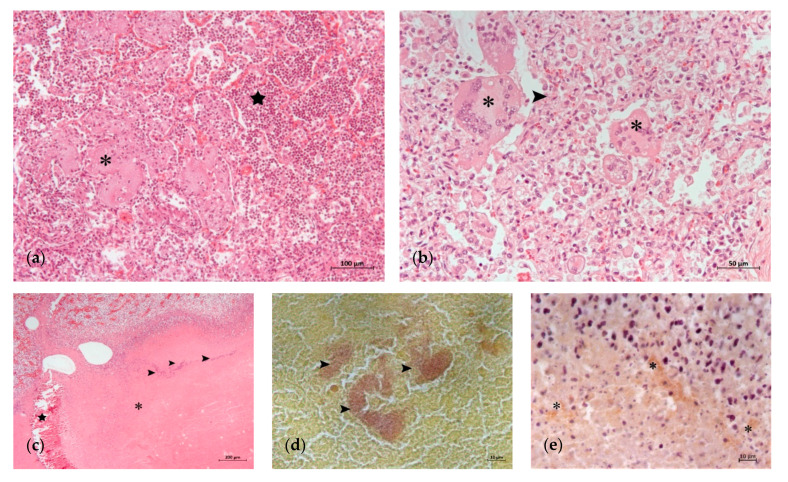
Lung lesions in which infection agents other than *M. bovis* are involved: (**a**) fibrinous bronchopneumonia with abundant fibrin (asterisk) and infiltrate of neutrophils in the alveolar lumens (star) (H-E); (**b**) interstitial pneumonia with thickening of alveolar septa (arrowhead) and syncytial cells (asterisks) (H-E); (**c**) extensive caseonecrotic focus (asterisk) with bacterial colonies inside (arrowheads) and areas of calcification (star) (H-E); (**d**) detail of Gram-positive bacterial colonies (arrowheads) in the caseonecrotic focus of (**c**); (**e**) detail of *M. bovis* antigen at the periphery of the caseonecrotic focus of (**c**) (asterisk) (IHC).

The histological lung lesions found in the 23 analyzed calves are summarized in Table 2. Lesions typically associated with *M. bovis*, such as caseonecrotic bronchopneumonia and bronchiolitis, were identified in 69.5 (16/23) and 65.2% (15/23) of the calves, respectively. These lesions were observed with different degrees of intensity and chronicity (Table 2, Figure 1 and Figure 2). Other lesions sometimes related to *M. bovis* such as necrosis of the bronchiolar epithelium, BALT hyperplasia and bronchiolar fibrosis, were identified in 52.1 (12/23), 65.2 (15/23) and 39.1% (9/23) of the animals, respectively (Table 2, Figure 1 and Figure 2). Lesions typically attributed to other bacteria were suppurative bronchopneumonia and fibrinous bronchopneumonia, which were identified in 73.9 (17/23) and 65.2% (15/23) of the calves, respectively (Table 2, Figure 3). Interstitial pneumonia, typically attributed to viruses, was identified in 47.8% (11/23) of the calves in addition to multinucleated syncytial cells, characteristic of BRSV (Table 2, Figure 3). Furthermore, Gram-positive and Gram-negative bacteria, and mineralization were detected in the necrotic foci of 34.7 (8/23), 26 (6/23) and 17.3% (4/23) of the calves, respectively (Table 2 and Figure 3).

Single or mixed pulmonary patterns were identified in all the animals (Table 3). Dual (39.1%; 9/23) and quadruple (30.4%; 7/23) concurrent patterns were the most frequently found, followed by single (17.3%; 4/23) and triple (13%; 3/23). Single patterns were caseonecrotic bronchopneumonia (8.6%; 2/23), fibrinous bronchopneumonia (4.3%; 1/23) and interstitial pneumonia (4.3%; 1/23) (Table 3).

IHC revealed the presence of *M. bovis* antigen in 73.9% (17/23) of the animals (Table 2). *M. bovis* antigen appeared in the caseonecrotic foci, most prominently at their periphery (Figure 2 and Figure 3), and adhered to the epithelial cells of the bronchiolar and bronchial lumens (Figure 1d). Among the IHC-positive animals, the most frequent lesions were caseonecrotic bronchopneumonia (94.1%; 16/17), bronchiolitis (88.2%; 15/17) and BALT hyperplasia (82.3%; 14/17). Other observed lesions were suppurative bronchopneumonia (76.4%; 13/17), necrosis of the bronchiolar epithelium (70.5%; 12/17), interstitial pneumonia and multinucleated syncytial cells (58.8%; 10/17), fibrinous bronchopneumonia (58.8%; 10/17) and bronchiolar fibrosis (52.9%; 9/17). In addition, Gram-positive (47%; 8/17) and Gram-negative bacteria (29.4%; 5/17), and mineralization (23.5%; 4/17) were observed in the necrotic foci (Table 2).

### 3.3. Antimicrobial Susceptibility of M. bovis Isolates

The MIC values for the reference strain PG45 were: tulathromycin, 1 µg/mL; tilmicosin, 0.5 µg/mL; lincomycin, 1 µg/mL; florfenicol, 4 µg/mL; gentamicin, 4 µg/mL; enrofloxacin, 0.125 µg/mL; marbofloxacin, 0.5 µg/mL; and oxytetracycline, 2 µg/mL.

The field isolates tested were recovered from lung (*n* = 9), nasal (*n* = 2), or auricular (*n* = 1) swabs. Individual MIC values for each isolate are shown in Table 1. MIC values were >128 µg/mL for tulathromycin, tilmicosin and lincomycin; 32 µg/mL for enrofloxacin; ≥8 µg/mL for oxytetracycline; and ≥4 µg/mL for florfenicol and gentamicin. MIC values were > 64 µg/mL for three of the four isolates tested against marbofloxacin and 0.5 µg/mL for the remaining isolate. These values reflected the low susceptibility of the isolates to the antimicrobials tested, with only two exceptions. One was the isolate from animal nº 20, recovered from the lung and susceptible to gentamycin (MIC = 4 µg/mL). Lesions typically attributed to *M. bovis* such as caseonecrotic bronchopneumonia or bronchiolitis and *M. bovis* antigen were observed in this animal (Table 2). The other exception was the isolate from animal nº 14, recovered from the nasal swab, and with a low MIC value for marbofloxacin (0.5 µg/mL) (Table 1). No lesions typically attributed to *M. bovis*, nor *M. bovis* antigen, were observed in this animal. Instead, lesions compatible with other bacteria such as fibrinous and suppurative bronchopneumonia were found (Table 2). Similar findings were observed in the animals nº 8 and nº 12, from which resistant isolates had been recovered from the lung and the auricular canal, respectively (Table 1 and Table 2). From the remaining eight animals, multiresistant isolates were recovered from the lung (*n* = 7) or the nasal (*n* = 1) swab. All of them were IHC-positive and presented at least one of the two lesions characteristic of *M. bovis* (Table 1 and Table 2).

## 4. Discussion

In this study, *M. bovis* was found in 86.9% of the feedlot calves (20/23) with clinical respiratory disease unresponsive to antimicrobial treatments. This value was comparatively higher than those reported in western (78.5%; 106/135) and eastern French calves (52.1%; 60/115) at the onset of BRD [4,16], and reinforces the hypothesis of a previous study sustaining that the infection may have become endemic in Spanish feedlot herds [28]. In that study, *M. bovis* was identified in 40.9% (84/205) of the beef cattle analyzed. More specifically, the pathogen was mainly detected in feedlot calves (81/183) and to a lesser extent in pasture-raised animals (3/22) housed in 26 different farms from five Spanish regions [28].

*M. bovis* was most often detected in the lungs (78.6%; 18/23) where it was found in a higher proportion than in northwestern Spain (66%; 33/50) and northern Italy (25%; 16/64), where only pneumonic lungs from asymptomatic animals were analyzed [5,15]. *M. bovis* was detected in other anatomical locations such as the nasal cavity (11/18), conjunctiva (6/18) and auricular canal (4/18), which reflect the capacity of the pathogen to disseminate in the host [36].

The *M. bovis* diagnosis in lung samples differed slightly depending on the technique used, with 73.9% (17/23) of positives by IHC and 65.2% (15/23) by culture and subsequent PCR. This difference may be because the diagnosis based on culture and PCR is limited by the viability of the mycoplasmas present in the sample. Only one animal (nº 8), showing suppurative and fibrinous bronchopneumonia, was found to be PCR-positive and IHC-negative. A possible explanation is that *M. bovis* was present and viable in the lung but in low concentration, so IHC failed to detect it. This hypothesis agrees with other authors observations who reported that IHC failed to detect small numbers of intralesional *M. bovis* organisms [5]. All in all, the combined use of the two methods augmented the sensitivity of the diagnosis.

Four pulmonary patterns were observed in the set of animals. Globally, caseonecrotic bronchopneumonia, typically attributed to *M. bovis*, was found in 69.5% (16/23) of the animals. Suppurative bronchopneumonia was observed in 73.9% (17/23) of the calves. This pattern is typically attributed to *P. multocida* and *T. pyogenes*, but some cases may be caused by *M. bovis* [12,37]. Fibrinous bronchopneumonia, typical of *M. haemolytica* and to a lesser extent *H. sommni*, was detected in 65.2% (15/23) of the calves [37]. Interstitial pneumonia, typical of viruses, and multinucleated syncytial cells, characteristic of BRSV, were identified in 47.8% (11/23) of the calves. This variety of morphological patterns was expected, as BRD is a complex entity characterized by various types of infection, leading to diverse histopathological lesions [8,12,17,18,19,20,21,22,37,38].

Given the multifactorial character of BRD, it is also common to find diverse patterns of pneumonic lesions in a single animal [21]. Our study was no exception as most of the calves (82.6%; 19/23) had a mixed pulmonary pattern, the quadruple combination of caseonecrotic, suppurative and fibrinous bronchopneumonia and interstitial pneumonia being the most frequent (30.4%; 7/23). In this context, viruses generally act as primary pathogens that, damaging the innate immunity and the epithelial surface of the airways, enable the participation of opportunistic bacteria. Among them, *M. bovis* can act as a primary pathogen [7]. However, this role is still controversial in some scientific communities and countries, mainly because this mycoplasma species is often found in asymptomatic carriers. Notably, caseonecrotic bronchopneumonia was the only pattern found in two animals (nº 20 and nº 22) and no Gram-positive nor Gram-negative bacteria were detected in the foci of necrosis. Although *M. bovis* was likely the primary cause, other bacteria, such as *M. haemolytica,* could have initiated the foci of necrosis and been removed by the antimicrobial therapy, as previously proposed [12]. Nevertheless, this finding still argues in favor of the role of *M. bovis* in respiratory disease. On the other hand, interstitial pneumonia and multinucleated syncytial cells were the only findings in one animal (nº 9). In effect, some viruses, such as BRSV and BHV-1, can induce life-threatening disease without bacterial superinfection [7,39]. Fibrinous bronchopneumonia was the only pattern found in another animal (nº 12), although no bacteria were observed in the foci of necrosis. They could have been eliminated by the antimicrobial treatment administered to the animal.

The involvement of *M. bovis* in the respiratory disease was confirmed by IHC in 73.9% (17/23) of the calves. In agreement with other studies [8,17,18,19,20,21], *M. bovis* antigen in the epithelial cells and surrounding caseonecrotic foci was related to the presence of caseonecrotic bronchopneumonia (94.1%; 16/17), bronchiolitis (88.2%; 15/17), BALT hyperplasia (82.3%; 14/17) and necrosis of the bronchiolar epithelium (70.5%; 12/17). Bronchiolar fibrosis, which has been also associated with *M. bovis*, was detected in 52.9% (9/17) of these animals and would indicate cases of greater chronicity [8]. Concurrent lesions typically caused by other bacteria and viruses were found in many *M. bovis* IHC-positive animals. Suppurative bronchopneumonia was detected in 76.4% (13/17) of these calves, and fibrinous bronchopneumonia and interstitial pneumonia with multinucleated syncytial cells were detected in 58.8% (10/17). These results are consistent with previous studies that reported different bacterial (*T. pyogenes*, *P. multocida*, *M. haemolytica* and *H. sommi)* and viral (BVDV, BHV-1, BRSV and PI-3) participants, in conjunction with *M. bovis,* in the development of BRD [4,5,6].

MIC values showed the low in vitro susceptibility of the *M. bovis* isolates (*n* = 12) to most of the antimicrobials received in vivo by the calves. This could be the result of resistance acquired after treatment [24,25]. Another possible explanation is that *M. bovis* isolates involved in the BRD episode were already resistant before any antimicrobial treatment, as recently observed in France [16]. Indeed, multiresistant strains currently circulate in that country, as it occurs in Spain [26,27,28]. Some authors propose that therapy in vivo may fail not because of the involvement of a resistant strain but because of the limited drug distribution into the caseous foci where *M. bovis* bacteria are most numerous [12]. This might explain the case of animal nº 20, with caseonecrotic bronchopneumonia and bronchiolitis, but a low MIC value for gentamicin (4 µg/mL). Nine of the 12 animals from which one resistant isolate was recovered had *M. bovis* antigen in lung lesions. The remaining three strains were recovered from animals without *M. bovis* antigen nor with lesions normally attributed to *M. bovis*. Given the multifactorial etiology of BRD, several variables may contribute to the clinical evolution of these animals, but still, the involvement of multiresistant *M. bovis* strains is likely contributing to the maintenance of the disease.

## 5. Conclusions

*M. bovis* plays a significant role in cases of clinical respiratory disease unresponsive to antimicrobial treatment that affects feedlots calves in Spain. The combined use of culture, PCR and IHC increases the sensitivity of *M. bovis* diagnosis in lung samples. Caseonecrotic bronchopneumonia is the morphological pattern most frequently observed in animals infected with *M. bovis*, and patterns indicative of other bacteria species and viruses can be concurrently detected. In some cases, *M. bovis* could have acted as the primary pathogen. *M. bovis* isolates recovered from animals with clinical respiratory disease are resistant in vitro to most of the antimicrobials specifically used for therapy in vivo.

## Figures and Tables

**Table 1:** Epidemiological background of the animals, anatomical location of *Mycoplasma bovis* and minimum inhibitory concentration (MIC) values.

Background				Anatomical Location of *M.bovis* ^3^					MIC (µg/mL) ^1^								
Animal ^1^	Country of Origin	Feedlot in Spain ^2^	Antimicrobial Treatment Received In Vivo	Ear	Conjunctiva	Nasal	Lung (Culture and PCR)	Lung (IHC)	Isolates Used for MIC Assays	Tul R ≥ 16	Tilm R ≥ 8	Lin R ≥ 8	Flor R ≥ 4	Gent R ≥ 8	Enr R ≥ 1	Marb R ≥ 1	Oxy R ≥ 4
1	France	RM-a	---	---	---	---	+	+	---	---	---	---	---	---	---	---	---
2	Spain	RM-b	---	---	---	---	+	+	---	---	---	---	---	---	---	---	---
**3**	Spain	VC-c	Flor, Sulfa, Amox	−	−	+	+	+	A150	---	---	---	8	---	---	---	---
**4**	Spain	VC-c	Flor, Sulfa, Amox	−	+	+	+	+	A156	---	---	---	8	---	---	---	---
5	Spain	VC-d	Amox	+	+	+	+	+	---	---	---	---	---	---	---	---	---
6	France	VC-d	Tilm, Oxy	−	−	−	−	+	---	---	---	---	---	---	---	---	---
7	Spain	VC-e	Tilm, Flor, Marb, Oxy, Amox	−	−	−	−	−	---	---	---	---	---	---	---	---	---
**8**	Spain	VC-e	Tilm, Flor, Marb	+	+	+	+	−	A203	---	>128	---	4	---	---	64	---
9	France	VC-d	Tilm, Flor, Dox	−	−	−	−	−	---	---	---	---	---	---	---	---	---
**10**	France	VC-d	Tilm, Flor, Oxy	−	−	−	+	+	A175	---	>128	---	4	---	---	---	8
**11**	France	VC-f	Tul, Tilm, Lin, Flor, Marb, Oxy	−	+	+	+	+	A160	>128	>128	>128	8	---	---	64	16
**12**	Spain	VC-c	Flor, Sulfa, Amox	+	−	−	−	−	A171	---	---	---	4	---	---	---	---
**13**	Spain	VC-e	Tilm, Enro, Oxy	−	−	+	−	+	A162	---	>128	---	---	---	32	---	16
**14**	France	VC-f	Tilm, Flor, Marb, Oxy, Amox	−	+	+	−	−	A168	---	>128	---	8	---	---	0.5	16
**15**	Spain	VC-g	Tul, Lin, Flor, Marb, Oxy, Amox	−	−	+	+	+	A215	>128	---	>128	8	---	---	64	32
16	Spain	VC-e	Tilm, Oxy, Amox	−	−	−	−	−	---	---	---	---	---	---	---	---	---
17	Spain	VC-h	---	−	−	+	+	+	---	---	---	---	---	---	---	---	---
**18**	France	VC-i	Flor	+	+	+	+	+	A223	---	---	---	32	---	---	---	---
**19**	France	VC-d	Tilm, Oxy, Amox	−	−	+	+	+	A219	---	>128	---	---	---	---	---	8
**20**	Romania	VC-j	Flor, Gent, Amox	−	−	−	+	+	A227	---	---	---	>128	4	---	---	---
21	Spain	RM-k	---	---	---	---	+	+	---	---	---	---	---	---	---	---	---
22	Romania	VC-l	---	---	---	---	+	+	---	---	---	---	---	---	---	---	---
23	Portugal	RM-b	---	---	---	---	−	+	---	---	---	---	---	---	---	---	---

--- = no data; + = positive; − = negative; RM = region of Murcia; VC = Valencian community; Flor = florfenicol; Sulfa = sulfadimidine; Amox = amoxicillin; Tilm = tilmicosin; Oxy = oxytetracycline; Marb = marbofloxacin; Dox = doxycycline; Tul = tulathromycin; Lin = lincomycin; Gent = gentamicin; PCR = polymerase chain reaction; IHC = immunohistochemistry; R = resistance breakpoint. ^1^ Numbers in bold indicate the animals from which one isolate was selected for MIC determination. MIC values were calculated for antimicrobials used for the treatment in vivo. MIC values for amoxicillin and sulfadimidine were not determined, as mycoplasmas are intrinsically resistant to these antimicrobials [25]. A single isolate per animal was tested; if possible, the isolate obtained from the lung swab was used for MIC assays. When no isolate was obtained from the lungs, MIC was determined for the isolate obtained from the nasal swab as the second option, or from the auricular swab as the third option. Resistance breakpoints encompasses the intermediate breakpoints. ^2^ Different letter (a-l) indicates different feedlot. ^3^ Anatomical location as detected by culture and PCR except in lungs, where both culture and PCR and IHC were carried out.

**Table 2:** Histopathological lung lesions and detection of *Mycoplasma bovis* by immunohistochemistry (IHC).

Animal	IHC	Caseonecrotic Bronchopneumonia ^1^	Suppurative Bronchopneumonia ^2^	Fibrinous Bronchopneumonia ^2^	Interstitial Pneumonia ^3^	Bronchiolitis ^4^	Bronchiolar Epithelial Necrosis ^5^	BALT Hyperplasia ^6^	Bronchiolar Fibrosis ^4^	Necrotic Foci ^7^
1	+	+	++	+	−	+	−	+	−	−
2	+	++	−	−	+	+	+++	+	++	−
3	+	++	++	+	+	+	++	+	+	G+/G−
4	+	++	+	+	+	++	−	+	+	G−
5	+	+	−	−	+	++	−	+++	++	−
6	+	+	+	+	+	++	++	+++	+	M
7	−	−	+	+	−	−	−	−	−	−
8	−	−	+	+	−	−	−	−	−	−
9	−	−	−	−	+	−	−	−	−	−
10	+	+	++	+	+	+	++	+	−	M, G+
11	+	+++	++	+	+	++	+++	++	+	M, G+
12	−	−	−	+	−	−	−	+	−	−
13	+	+	+	−	−	+	−	+	++	−
14	−	−	+	+	−	−	−	−	−	G−
15	+	−	+++	++	−	+	+	+	−	G+/ G−
16	−	−	+	+	−	−	−	−	−	−
17	+	+	++	+++	+	−	+	−	−	G+/G−
18	+	+	++	+	−	−	+	−	−	G+
19	+	++	++	+	+	+	+	+	−	G+/G−
20	+	+	−	−	−	+	−	−	−	−
21	+	++	+++	−	+	++	++	+++	+	M, G+
22	+	+++	−	−	−	+	+++	++	++	−
23	+	+	+	−	−	++	++	+	−	−

BALT = bronchus-associated lymphoid tissue; − = negative; G+ = Gram-positive bacteria; G− = Gram-negative bacteria; M = mineralization; ^1^ + size of foci < 200 µm; ++ size of foci >200 µm, < 2000 µm; +++ size of foci > 2000 µm. **^2^** + < 25%; ++ > 25%, < 75%; and +++ >75%. ^3^ + presence of syncytial cells. ^4^ + mild, ++ moderate, +++ severe. ^5^ + < 10%; ++ > 10%, < 50%; +++ 50%). ^6^ + 1–2 lymphoid follicles < 100 µm, ++ 1–2 lymphoid follicles > 100, +++ > 2 lymphoid follicles. ^7^ Necrotic foci refers to foci of coagulative necrosis, abscesses and foci of caseous necrosis.

**Table 3:** Combination of pulmonary patterns.

Patterns of Pneumonic Lesions	Number of Animals	List of Animals
CB, SB, FB, IP	7	3, 4, 6, 10, 11, 17, 19
CB, SB, FB	2	1, 18
CB, SB, IP	1	21
CB, SB	2	13, 23
CB, IP	2	2, 5
SB, FB	5	7, 8, 14, 15, 16
CB	2	20, 22
FB	1	12
IP	1	9

CB = caseonecrotic bronchopneumonia; SB = suppurative bronchopneumonia; FB = fibrinous bronchopneumonia; IP = interstitial pneumonia.

## Data Availability

The data presented in this study are available on request from the corresponding author.
